# Meyerson Phenomenon Over Nuchal Nevus Simplex

**DOI:** 10.5826/dpc.1101a98

**Published:** 2020-12-07

**Authors:** Ada Claret-de Castro, Marc Mir-Bonafé, José María Mir-Bonafé, Juan Francisco Mir-Bonafé

**Affiliations:** 1 Department of Pediatrics, Hospital General de Granollers, Barcelona, Spain; 2 Department of Dermatology, Hospital Universitario Central de Asturias, Oviedo, Spain; 3 Department of Dermatology, Clínica Juaneda, Palma de Mallorca, Spain; 4 Department of Dermatology, Hospital Son Llàtzer, Palma de Mallorca, Spain

**Keywords:** Meyerson phenomenon, nevus simplex, salmon patch, capillary malformation, eczematous changes

## Case Presentation

**Figure 1:**
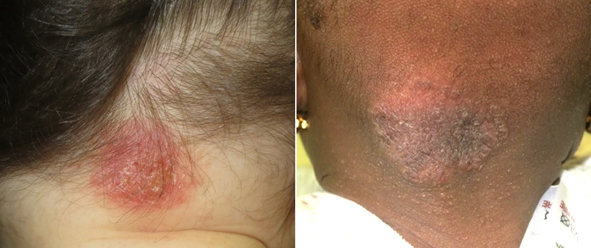
Two cases of Meyerson phenomenon over nuchal nevus simplex.

Two infants aged 11 and 5 months consulted for eczematous plaques on the nape. Their parents reported that the lesions had developed over previous erythematous stains. Both cases corresponded to eczematous changes over nevus simplex, known as Meyerson phenomenon (Figure 1).

## Teaching Point

Meyerson phenomenon is defined as a spontaneous eczematous reaction within an overlying skin lesion. This phenomenon was first reported in melanocytic lesions [1]; however, it has also been related to seborrheic keratosis, dermatofibroma, molluscum contagiosum, and many other lesions. Although many hypotheses have been proposed, its origin is still unknown, and its relationship with atopic dermatitis remains controversial. Even though Meyerson phenomenon over capillary malformations is not frequent, it has been associated with nevus simplex [2,3], port-wine stains [4], and trunk and limb capillary malformations [5]. Exceptionally, it has been reported after laser treatment [4]. Although its diagnosis is clinical, in doubtful cases we may perform histopathological study. Histopathological findings include characteristic features of eczema such as spongiosis, acanthosis, parakeratosis, and superficial dermal lymphocytic infiltrate overlying capillary ectasias [1,5]. Prognosis is good, and topical corticosteroids are usually successfully used to treat the eczema. Nevertheless, some lesions may recur after treatment discontinuation, and pulsed dye laser therapy sometimes may be needed [2,5].
